# Intrinsic resistance to PIM kinase inhibition in AML through p38α-mediated feedback activation of mTOR signaling

**DOI:** 10.18632/oncotarget.9822

**Published:** 2016-06-05

**Authors:** Diede Brunen, María José García-Barchino, Disha Malani, Noorjahan Jagalur Basheer, Cor Lieftink, Roderick L. Beijersbergen, Astrid Murumägi, Kimmo Porkka, Maija Wolf, C. Michel Zwaan, Maarten Fornerod, Olli Kallioniemi, José Ángel Martínez-Climent, René Bernards

**Affiliations:** ^1^ Division of Molecular Carcinogenesis, The Netherlands Cancer Institute, Amsterdam, The Netherlands; ^2^ Division of Oncology, University of Navarra, Pamplona, Spain; ^3^ Institute for Molecular Medicine Finland (FIMM), University of Helsinki, Helsinki, Finland; ^4^ Department of Pediatric Oncology, Erasmus Medical Center/Sophia Children's Hospital, Rotterdam, The Netherlands; ^5^ Hematology Research Unit Helsinki, Department of Medicine, Helsinki University Central Hospital and University of Helsinki, Helsinki, Finland

**Keywords:** AML, PIM, AZD1208, p38, resistance

## Abstract

Although conventional therapies for acute myeloid leukemia (AML) and diffuse large B-cell lymphoma (DLBCL) are effective in inducing remission, many patients relapse upon treatment. Hence, there is an urgent need for novel therapies. PIM kinases are often overexpressed in AML and DLBCL and are therefore an attractive therapeutic target. However, *in vitro* experiments have demonstrated that intrinsic resistance to PIM inhibition is common. It is therefore likely that only a minority of patients will benefit from single agent PIM inhibitor treatment. In this study, we performed an shRNA-based genetic screen to identify kinases whose suppression is synergistic with PIM inhibition. Here, we report that suppression of p38α (MAPK14) is synthetic lethal with the PIM kinase inhibitor AZD1208. PIM inhibition elevates reactive oxygen species (ROS) levels, which subsequently activates p38α and downstream AKT/mTOR signaling. We found that p38α inhibitors sensitize hematological tumor cell lines to AZD1208 treatment *in vitro* and *in vivo*. These results were validated in *ex vivo* patient-derived AML cells. Our findings provide mechanistic and translational evidence supporting the rationale to test a combination of p38α and PIM inhibitors in clinical trials for AML and DLBCL.

## INTRODUCTION

Cancer cells are highly dependent on oncogenic signaling, making targeted agents that specifically inhibit these pathways an attractive treatment option. Unfortunately, the majority of inhibitors demonstrate a modest or complete lack of response when used as a single agent, due to rapid resistance caused by redundancy or feedback signaling [1]. As a result, many potentially useful agents will not make it through early phase clinical trials. It is therefore essential, even before the start of clinical studies, to identify powerful drug combinations that prevent therapy resistance. One such promising class of drugs currently being evaluated in phase I clinical trials are the PIM kinase inhibitors (NCT01588548, NCT01489722, NCT02078609, and NCT02160951).

PIM kinases (PIM1, −2, and −3) are a family of short-lived, constitutively active serine/threonine kinases which are often overexpressed in hematological tumors, including acute myeloid leukemia (AML), chronic myeloid leukemia (CML), the activated B-like (ABC) subtype of diffuse large B-cell lymphoma (DLBCL), and multiple myeloma (MM) [2–6]. The expression of PIM kinases is largely regulated *via* the JAK/STAT pathway on a transcriptional level, since PIM kinases have a short half-life [7–9]. Approximately 30 percent of AML patients harbor the FLT3 internal tandem duplication (FLT-ITD), which results in high JAK/STAT signaling and consequently PIM overexpression [10–12]. Furthermore, the transcription factors NF-κB and HOXA9 can regulate PIM expression and are often highly active in AML [13–15].

PIM kinases are highly redundant and regulate the activity of substrates involved in translation, survival, cell cycle, and MYC-dependent transcription. Several substrates overlap with AKT/mTOR signaling, including PRAS40 [16], TSC2 [5], 4EBP1 [17], and EIF4B [18–20]. Furthermore, PIM kinases can suppress apoptosis by phosphorylating BCL2-associated agonist of cell death (BAD) [21–23], resulting in the dissociation of BCL-2 and BCL-XL. Other substrates include MYC, and the cell cycle regulating proteins p21 (CDKN1A) [24, 25], p27KIP1 (CDKN1B) [26], CDC25A [27], and CDC25C [27].

PIM kinases contain a unique ATP-binding pocket, which has resulted in the development of highly selective pan-PIM inhibitors such as AZD1208 [6, 28, 29]. However, *in vitro* experiments have already demonstrated intrinsic resistance to PIM inhibitors, which will likely undermine the success of these compounds in the clinic [6, 30]. Indeed, initial phase I clinical trial results demonstrate no benefit from the use of AZD1208 in advanced solid tumors and malignant lymphoma (NCT01588548). In this study, we used an shRNA-based screening approach to identify kinases whose suppression is synergistic with PIM inhibition.

## RESULTS

### An shRNA screen identifies *p38α* loss to enhance the efficacy AZD1208

**Figure 1:**
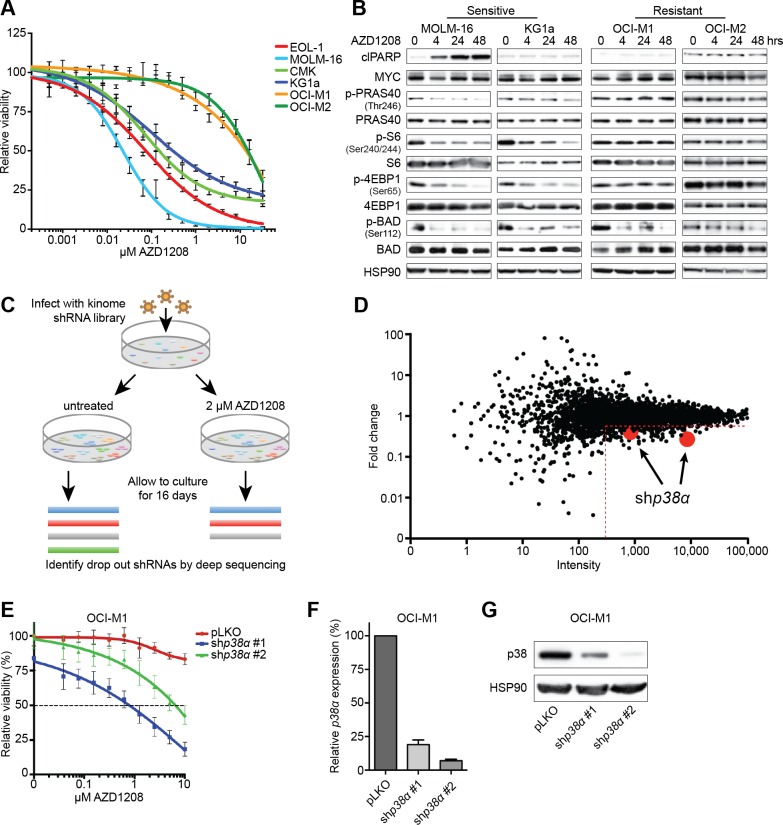
An shRNA screen identifies *p38α* loss to be synthetic lethal with PIM inhibitor treatment **A.** Short-term viability assay of a panel of AML cell lines. Cells were treated for 5 days with increasing concentrations of AZD1208 and viability was measured using CellTiter-Blue (*n* = 3). **B.** AZD1208-resistant AML cell lines demonstrate sustained mTOR signaling upon treatment. Cells were treated for 4, 24, and 48 hours with 1 μM AZD1208 followed by western blot analysis (*n* = 3). **C.** Schematic outline of the synthetic lethality screen. OCI-M1 cells were infected with a lentiviral kinome shRNA library and cultured in the presence or absence of 2 μM AZD1208 for 16 days in replicates. shRNA barcodes were subsequently recovered by PCR and analyzed by next generation sequencing. **D.** The shRNA screen identifies *p38α* loss to be synthetic lethal with AZD1208. The x-axis depicts the average number of sequencing reads in the untreated sample (intensity). The y-axis depicts the fold change in abundance of shRNAs in the treated *versus* untreated population. Two shRNAs (> 300 reads) targeting *p38α* were depleted in the treated population with a fold change of > 2. **E.** *p38α* knock down enhances AZD1208 response. OCI-M1 cells were infected with two shRNAs targeting *p38α* - pLKO serves as the control - and treated for 5 days with AZD1208. Cell viability was measured using CellTiter-Blue (*n* = 3). **F.-G.** Knock down of *p38α* was assessed on RNA and protein level.

To study the effect of PIM inhibition on AML cell growth, we treated 6 cell lines expressing PIM1 or PIM2 for 5 days with AZD1208 and measured viability (Figure 1A and S1A). Whereas 4 out of 6 cell lines were sensitive to treatment (IC_50_ < 0.5 μM), 2 cell lines (OCI-M1 and OCI-M2) were intrinsically resistant to AZD1208 (IC_50_ > 10 μM). To gain insights into the biochemical differences underlying these responses, we treated 2 sensitive and 2 resistant cell lines with AZD1208 and analyzed the major targets of PIM kinases (Figure 1B). We observed a complete blockade of BAD phosphorylation in all cell lines, indicating that AZD1208 inhibited PIM kinase activity. In contrast, only the sensitive cell lines demonstrated reduced phosphorylation of PRAS40, S6, and 4EBP1. Since these targets are also regulated by mTOR, resistance might occur through sustained mTOR signaling upon PIM inhibition.

To identify genes whose suppression confers sensitivity to AZD1208, we performed a loss of function genetic screen using a kinome library consisting of 3530 short hairpins RNAs (shRNA) in lentiviral vectors targeting 535 kinases and kinase-related genes [31]. We infected OCI-M1 cells with the library and cultured cells in the presence or absence of 2 μM AZD1208 (Figure 1C). After 16 days, we isolated genomic DNA, recovered the shRNA inserts by PCR amplification, and performed next generation sequencing to quantify the shRNA sequences. The individual replicates highly correlated and clustered together according to the absence or presence of drug (Figure S1B-S1C). To exclude false positives, we only called genes a hit when at least 2 shRNAs - present with more than 300 reads in the untreated population - gave a minimal fold-reduction of 2. Furthermore, these shRNAs should not be depleted from the untreated population compared to the T_0_ sample - indicative of a straight lethal effect. Two hairpins targeting mitogen activated protein kinase 14 (*MAPK14* or *p38α*) fulfilled these criteria, suggesting that *p38α* loss sensitizes AML cells to PIM inhibition (Figure 1D). We validated the shRNAs targeting *p38α* by treating drug resistant parental OCI-M1 cells or *p38α* knockdown cells with AZD1208 in a 5-day viability assay (Figure 1E). The level of knockdown was assessed on protein and mRNA level (Figure 1F-1G).

### Pharmacological inhibition of p38α restores sensitivity to AZD1208 through inhibition of mTOR signaling

**Figure 2:**
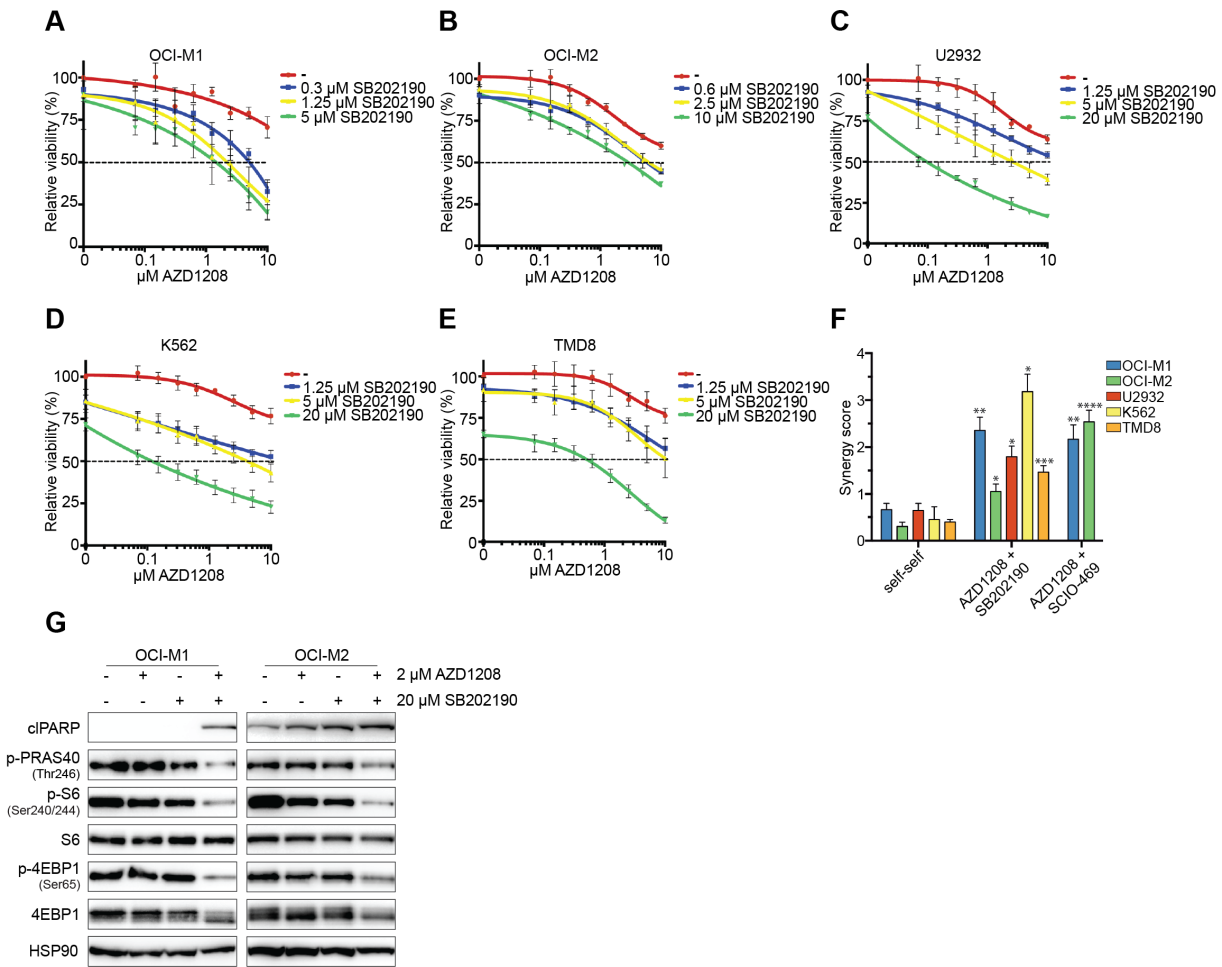
Pharmacological inhibition of p38 synergizes with AZD1208 through reduced mTOR signaling **A.-E.** p38 inhibitors enhance AZD1208 response. OCI-M1, OCI-M2, U2932, K562, and TMD8 cells were treated with increasing concentrations of AZD1208 (x-axis) and co-treated with the p38 inhibitor SB202190. Viability was measured after 5 days using CellTiter-Blue (*n* = 3). **F.** p38 inhibitors are synergistic with AZD1208. OCI-M1, OCI-M2, U2932, K562, and TMD8 cells were treated with 2-fold dilutions of AZD1208, SB202190, SCIO-469, or combinations for 5 days. Viability was assessed by CellTiter-Blue and used to calculate synergy scores. Self-self combination treatments were used as a baseline to determine significance (*n* = 3). P-values were calculated using a one-way ANOVA and Dunnett's test. *p* ≤ 0.05 (*), *p* ≤ 0.01 (**), *p* ≤ 0.001 (***), and *p* ≤ 0.0001 (****) **G.** Combined inhibition of PIM and p38 results in reduced AKT/mTOR signaling after 48 hours. OCI-M1 (2.10^5^ cells/well in 6-well plate) and OCI-M2 (4.10^5^ cells/well in 6-well plate) cells were treated for 48 hours with 2 μM AZD1208, 20 μM SB202190, or the combination. Cell lysates were harvested and subjected to western blot analysis (*n* = 3).

To assess whether AZD1208 synergizes with p38 inhibitors, we treated resistant AML (OCI-M1, OCI-M2), DLBCL (U2932, TMD8), and chronic myeloid leukemia (CML) (K562) cell lines with the combination of AZD1208 and the p38 inhibitors SB202190 or SCIO-469 (the latter is in phase II clinical trial for multiple myeloma: NCT00095680) (Figure 2A-2E and Figure S2A-S2C). Single drug treatment modestly inhibited viability, whereas dual inhibition synergistically suppressed growth. To determine the level of synergy, we used a previously described method that calculates additive effects (based on the Loewe smodel) and subtracts these values from the experimental values resulting in a synergy score (Figure S2D) [32]. We furthermore calculated combination indices - which is a widely accepted method to calculate synergy. A high synergy score and vice versa a low combination index indicate strong synergy. To determine the significance of our findings, we also treated cells with self-self inhibitor combinations, which theoretically should result in a synergy score of 0 and a combination index of 1. As expected, combined treatment of AZD1208 and either SB202190 or SCIO-469 resulted in high synergy scores and low combination indices (Figure 2F and Figure S2E).

Since AZD1208 treatment did not suppress mTOR signaling in resistant cell lines, we analyzed this pathway upon dual PIM/p38 inhibition. Combined treatment reduced p-PRAS40, p-S6, and p-4EBP1 levels in all cell lines after 48 hours (Figure 2G and Figure S2F). In addition, both AZD1208-resistant and sensitive cell lines were sensitive to mono-treatment with the mTOR inhibitor AZD8055, suggesting that the growth inhibitory effect of AZD1208 is mainly *via* its effects on mTOR signaling (Figure S2G). Together, these data suggest that p38 inhibition can block PIM inhibitor resistance through suppression of mTOR signaling.

### PIM inhibition activates p38/AKT signaling

**Figure 3:**
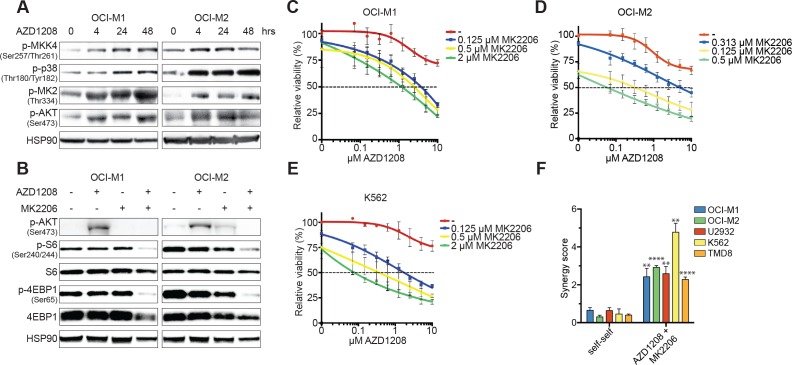
Activation of the p38 pathway upon PIM inhibition **A.** The p38 signaling pathway is activated upon AZD1208 treatment. OCI-M1 (2.10^5^ cells/well in 6-well plate) and OCI-M2 (4.10^5^ cells/well in 6-well plate) cells were treated with 2 μM AZD1208 for 4, 24, and 48 hours. Cell lysates were harvested and subjected to western blot analysis (*n* = 3). **B.** Dual PIM/AKT inhibition synergistically inhibits mTOR signaling. OCI-M1 (2.10^5^ cells/well in 6-well plate) and OCI-M2 (4.10^5^ cells/well in 6-well plate) cells were treated for 48 hours with 2 μM AZD1208, 2 μM MK2206, or the combination. Cell lysates were harvested and subjected to western blot analysis (*n* = 3). **C.-E.** AKT inhibition enhances AZD1208 response. OCI-M1, OCI-M2, and K562 cells were treated with increasing concentrations of AZD1208 (x-axis) and co-treated with the AKT inhibitor MK2206. Viability was measured after 5 days using CellTiter-Blue (*n* = 3). **F.** MK2206 is synergistic with AZD1208. OCI-M1, OCI-M2, U2932, K562, and TMD8 cells were treated with 2-fold dilutions of AZD1208, MK2206, or the combination for 5 days. Viability was assessed by CellTiter-Blue and used to calculate synergy scores. Self-self combination treatments were used as a baseline to determine significance (*n* = 3). P-values were calculated using a one-way ANOVA and Dunnett's test. *p* ≤ 0.01 (**) and *p* ≤ 0.0001 (****)

To gain further mechanical insights in the synergy between p38 and PIM inhibition, we treated AML cell lines with AZD1208 and assessed the activity of p38 signaling (Figure 3A). PIM inhibition elevated phosphorylation levels of p38, MKK4 (upstream kinase), and MK2 (downstream target of p38). Milder activation of p38 and MK2 was observed in DLBCL and CML cell lines (Figure S3A). Several reports have described that MK2 - the downstream target of p38 - acts an activator of AKT by enhancing phosphorylation of serine 473 and 308, although the exact mechanism remains unclear [33–35]. Since resistant cell lines demonstrated sustained mTOR signaling upon AZD1208 treatment, we investigated whether PIM inhibition could activate mTOR *via* AKT. As depicted in Figure 3A, cell lines activate AKT upon AZD1208 treatment. Furthermore, combined treatment with AZD1208 and the AKT inhibitor MK2206 synergistically blocked mTOR signaling and cell growth in a comparable manner to p38 inhibition (Figure 3B-3F and Figure S3B-S3D).

### AZD1208 treatment elevates ROS levels

**Figure 4:**
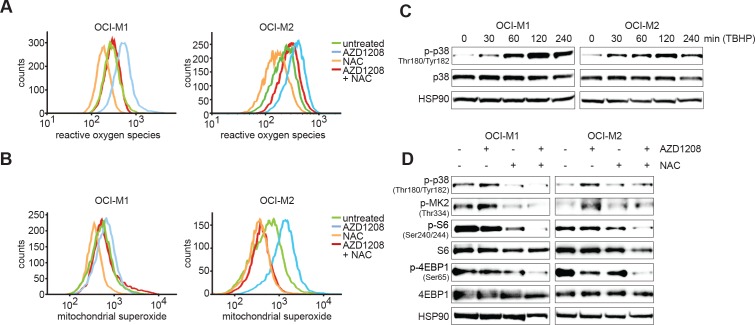
PIM inhibition elevates ROS and mitochondrial superoxide levels **A.-B.** OCI-M1 and OCI-M2 cells were treated for 48 hours with 5 μM AZD1208, 5 mM NAC, or the combination. Cells were incubated with CellRox or MitoSox - according to the manufacturer's instructions - to stain for respectively ROS or mitochondrial superoxide and fluorescent intensity was measured by flow cytometry analysis (*n* = 3). **C.** Induction of ROS activates p38. OCI-M1 and OCI-M2 cells were treated for 0, 30, 60, 120, or 240 min with 400 μM TBHP. Cell lysates were harvested and subjected to western blot analysis (*n* = 3). **D.** ROS inhibition prevents PIM inhibitor-mediated feedback activation of p38. OCI-M1 (2.10^5^ cells/well in 6-well plate) and OCI-M2 (4.10^5^ cells/well in 6-well plate) cells were treated for 48 hours with 2 μM AZD1208, 5 mM NAC, or the combination. Cell lysates were harvested and subjected to western blot analysis (*n* = 3)

We subsequently investigated the mechanism by which PIM inhibition results in p38 activation. A recent study by Song *et al*. demonstrated that PIM knockout results in elevated reactive oxygen species (ROS) levels in mouse embryonic fibroblasts (MEFs) [36]. Since ROS is a known activator of p38 signaling [37], we measured total ROS and mitochondrial superoxide levels upon AZD1208 treatment in OCI-M1 and OCI-M2 cell lines (Figure 4A-4B). Interestingly, both ROS and superoxide levels were increased upon PIM inhibition, which could be reverted by treating cells with the ROS scavenger N-acetyl-cysteine (NAC). To validate that increased ROS levels can activate p38 in our cell lines, we treated OCI-M1 and OCI-M2 cell lines with ROS-inducing tert-butyl hydrogen peroxide (TBHP). As expected, induction of ROS levels results in rapid activation of p38 (Figure 4C). Furthermore, NAC prevented the activation of p38 signaling upon AZD1208 treatment and blocked activation of S6/4EBP1 (Figure 4D). These results suggest that PIM-inhibitor mediated upregulation of ROS might induce therapy resistance by activation of p38/AKT signaling.

### Pharmacological inhibition of p38α enhances the efficacy of AZD1208 in primary AML cells and mouse xenografts

**Figure 5:**
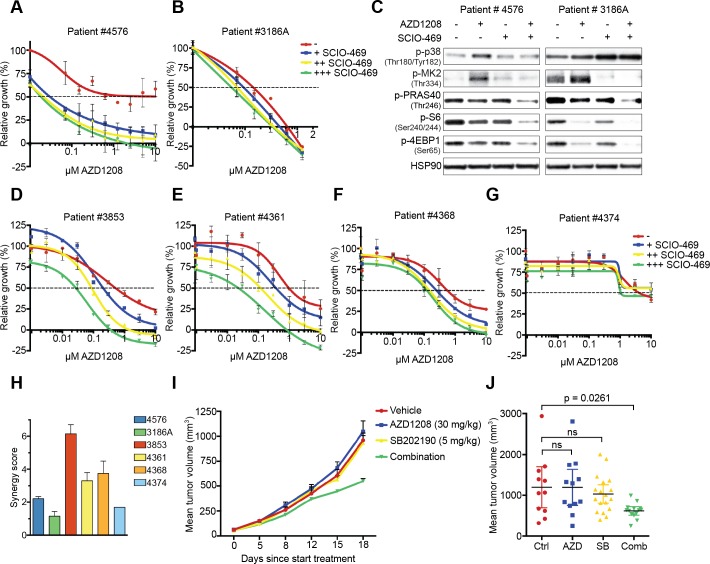
Suppression ­­­of p38 signaling restores sensitivity to PIM inhibition in primary AML cells and mouse xenografts **A.-B.** Inhibition of p38 enhances AZD1208 response in primary AML cells. Patient cells were treated with increasing concentrations of AZD1208 (x-axis) and co-treated with 0.313 μM, 1.25 μM, or 5 μM SCIO-469. Growth was measured after 4 days using MTT. A day 0 measurement was used as a baseline value (y-axis = ‘0’) for growth. Negative relative growth indicates a cytotoxic effect (*n* = 2). **C.** Combined p38/PIM inhibition suppresses mTOR signaling. Primary AML cells were treated for 24 hours with 1 μM AZD1208, 10 μM SCIO-469, or the combination. Cell lysates were harvested and subjected to western blot analysis. **D.-G.** Inhibition of p38 enhances AZD1208 response in a validation set of primary AML cells. Cells were treated with increasing concentrations of AZD1208 (x-axis) and co-treated with 0.3 μM, 1 μM, or 3 μM SCIO-469. Viability was measured after 72 hours using CellTiter-Glo. A day 0 measurement was used as a baseline value (y-axis = ‘0’) for growth. Negative relative growth indicates a cytotoxic effect (*n* = 2). **H.** SCIO-469 is synergistic with AZD1208. Primary AML cells were treated with 2-fold dilutions of AZD1208, SCIO-469, or the combination. Viability was assessed by CellTiter-Blue and used to calculate synergy scores. **I.** Dual PIM/p38 inhibition suppresses tumor growth in a xenograft model. K562 cells (5.10^6^) were subcutaneously implanted in Rag2^−/−^IL2γc^−/−^ mice. Once tumors were established, animals were treated with vehicle, AZD1208 (30 mg/kg), SB202190 (5 mg/kg), or both drugs in combination. **J.** Mean tumor volume after 18 days treatment. Vehicle (*n* = 11), AZD1208 (*n* = 12), SB202190 (*n* = 18), and combination (*n* = 14). *P*-values were calculated *via* one-way ANOVA and Dunnett's test.

To translate our findings into a possible combination therapy in the clinic, we performed *ex vivo* drug testing assays with PIM-expressing AML patient-derived mononuclear cells obtained from the Erasmus Medical Center (Rotterdam, The Netherlands). Unfortunately, only 2 out of 7 available samples proliferated *ex vivo,* and were therefore considered for further experiments (Figure S4A). Since this particular drug combination mainly acts cytostatic rather than cytotoxic, we used the T_0_ value as a baseline (y-axis = ‘0’) for viability in the presented graphs rather than phenyl arsenic oxide (PAO). As a result, negative relative growth indicates a cytotoxic effect. Combined p38/PIM inhibitor treatment reduced proliferation in both AZD1208 resistant (#4576) and sensitive (#3186A) patient samples (Figure 5A-5B). Furthermore, dual p38/PIM inhibition prevented feedback activation of mTOR signaling in these cells (Figure 5C).

To extend these findings, we tested an additional 7 AML patient samples derived from the Helsinki University Central Hospital (Helsinki, Finland). Five of these samples proliferated *ex vivo*, and were therefore considered for further experiments (Figure S4B). Combined p38/PIM inhibitor treatment reduced cell growth in 4 out of 5 samples (#3853, #4361, # 4368, and #4374) (Figure 5D-5G and Figure S4C). We subsequently performed a synergy analysis on all 6 samples (both Rotterdam and Helsinki data sets) (Figure 5H and Figure S4D). We observed high synergy scores and low combination indices in 4 of the 6 samples tested, suggesting that the combination of p38/PIM inhibitors is potentially beneficial for the treatment of AML.

Our *in vitro* and *ex vivo* data suggest that PIM and p38 inhibition should also be synergistic when combined *in vivo*. To test this, we xenografted K562 cells into immunodeficient mice, treated animals with vehicle, AZD1208, SB202190, or the combination and assessed tumor growth (Figure 5I-5J). Single agent treatment did not inhibit tumor growth, whereas again only the combined treatment significantly reduced growth.

## DISCUSSION

**Figure 6:**
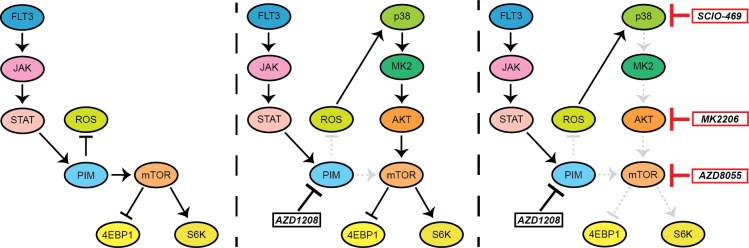
Schematic overview of feedback activation of mTOR signaling upon PIM inhibition AZD1208 treatment inhibits PIM activity; thereby elevating ROS levels which subsequently results in resistance through activation of p38, AKT, and mTOR signaling. Combined treatment of AZD1208 with either a p38 inhibitor (SCIO-469) or an AKT inhibitor (MK2206) prevents feedback activation of mTOR and restores PIM inhibitor sensitivity. Single mTOR inhibitor treatment (AZD8055) also prevents cell growth, indicating the dependency of hematopoietic tumor cells to this pathway.

Novel therapies are of utmost importance for the treatment of AML and DLBCL, since many patients relapse with conventional therapies [38, 39]. PIM kinases are often overexpressed and represent an interesting target due to the availability of highly selective inhibitors. Furthermore, mice deficient for all PIM kinases have no significant phenotype, which may indicate a favorable toxicity profile of specific PIM inhibitors *in vivo* [40]. However, similar to other targeted agents, resistance to PIM inhibitors occurs. This study provides a rationale to test a combination of p38 and PIM inhibitors in clinical trials. We demonstrated that resistance to AZD1208 occurs through feedback activation of mTOR signaling, which is mediated by ROS, p38, and AKT (Figure 6).

A recent study by Song *et al*. demonstrated elevated ROS levels upon PIM loss in mouse embryonic fibroblast cells [36]. Triple knock-out cells for PIM1, −2, and −3 were found to have decreased expression of genes involved in mitochondrial oxidative phosphorylation and lower levels of the ROS detoxifying genes superoxide dismutase (SOD) 1, −2, and −3, glutathione peroxidase 4 (GPX4), and peroxiredoxin 3 (PRDX3). Another study by Lilli *et al*. describes that PIM expression delays mitochondrial dysfunction and ROS production upon IL-3 starvation in the murine myeloid cell line FDCP1 [41]. However, these findings were not the result of reduced SOD activity, but suggested to be through elevated expression of BCL-2. This gene mainly plays a role in apoptosis, but also modulates oxidative phosphorylation and ROS levels [42, 43]. Interestingly, several groups have reported a synergistic interaction between PIM and BCL-2 inhibitors in hematological and solid tumors [44, 45].

Our results are in concordance with previous findings showing synergy between PIM and PI3K or AKT inhibitors [30, 46]. However, these studies lacked insights in the mechanism underlying this synergistic interaction. Even more important, p38 inhibitors - which are in clinical trial for diseases such as rheumatoid arthritis, chronic obstructive pulmonary disease, and multiple myeloma - are likely less toxic than PI3K, AKT, and mTOR inhibitors and could therefore be preferred for combination therapies. Lower toxicity is particularly important since these targeted agents will likely be used on top of a chemotherapeutic backbone. To conclude, our findings suggest that AML patients might benefit from combined PIM and p38 inhibitor treatment.

## MATERIALS AND METHODS

### Cell culture and lentiviral transduction

OCI-M1 and OCI-M2 cells were cultured in Iscove's Modified Dulbecco's Medium (IMDM). EOL-1, CMK, MOLM-16, KG1a, TMD8, U2932, and K562 cells were cultured in Roswell Park Memorial Institute (RPMI) 1640. Medium was supplemented with 16% fetal calf serum (FCS), 1% glutamine, and 1% penicillin/streptomycin, and cells were grown at 37°C and 5% CO_2_. All cells were purchased at DSMZ, except TMD8, which was a kind gift from AstraZeneca, and K562, which was available within The Netherlands Cancer Institute. Cultured cells were routinely tested for mycoplasma infection.

PEI transfection of HEK293T cells was used to produce lentiviral supernatant. Target cells were seeded 1 day before infection. Lentivirus was added to the medium in the presence of 5 μg/ml polybrene. Cells were selected for successful lentiviral integration using 2 μg/ml puromycin. All lentiviral shRNA vectors were retrieved from the arrayed TRC human genome-wide shRNA collection. For individual knock down of genes, the following hairpins were used:

*p38α* _TRCN0000000513_CCATTTCAGTCCATCATTCAT;

*p38α_*TRCN0000010052_GTTCAGTTCCTTATCTACCAA;

Control infections were performed with the empty pLKO.1 vector.

### Synthetic lethality screen

A kinome library - consisting of 3530 short hairpins RNAs (shRNA) in lentiviral vectors, targeting 535 kinases and kinase-related genes - was used to infect OCI-M1 cells with 1000-fold coverage. Cells were cultured in the absence or presence of drug in biological replicates for 16 days, followed by genomic DNA isolation using the DNAeasy Blood & Tissue Kit (Qiagen). shRNA cassettes were amplified using Phusion High Fidelity DNA polymerase (Thermo Scientific) by performing a 2 step PCR amplification: (1) 98°C, 30 s; (2) 98°C, 10s; (3) 60°C, 20 s; (4) 72°C, 1 min; (5) to step 2, 16 cycles; (6) 72°C, 5 min; (7) 4°C. The abundance of each hairpin in the treated *versus* untreated pools was determined by Illumina next generation sequencing.

### Protein lysate preparation and western blot analysis

Cells were lysed using RIPA buffer containing 150 mM NaCl, 50 mM Tris pH 8.0, 1% NP-40, 0.5% sodium deoxycholate and 0.1% SDS supplemented with protease inhibitors (Complete, Roche) and phosphatase inhibitor cocktails II and III (Sigma). Sample buffer (60mM Tris pH 6.8, 5% glycerol, 1% SDS, 2% β-mercaptoethanol, 0.02% bromophenol blue) was added, lysates were boiled for 10 minutes, and equal amounts of sample were subjected to SDS gel electrophoresis followed by western blotting. Primary antibody against HSP90 (SC-7947) was purchased from Santa Cruz. Antibodies against clPARP (#5625), PIM1 (#3247), PIM2 (#4730), p-AKT (#4060), AKT (#2920), p-PRAS40 (#2997), PRAS40 (#2610), p-S6 (#2215), S6 (#2217), p-4EBP1 (#9456), 4EBP1 (#9452), p-BAD (#5284), BAD (#9292), p-p38 (#4511), p38 (#8690), p-MK2 (#3007), MK2 (#3042), and p-MKK4 (#9156) were from Cell Signaling. Secondary antibodies were obtained from Bio-Rad Laboratories. AZD1208 was a kind gift from AstraZeneca. SCIO-469 was purchased from Tocris, SB202190 from Selleckchem, and NAC from Sigma.

### Quantitative RT-PCR

RNA was isolated using a Quick-RNA MiniPrep kit (Zymo Research, #R1055). Subsequent cDNA synthesis was performed using Maxima Universal First Strand cDNA Synthesis Kit (Thermo scientific, # K1661).

The 7500 Fast Real-Time PCR System from Applied Biosystems was used to measure mRNA levels. mRNA expression levels were normalized to expression of *GAPDH*. The following primer sequences were used in the SYBR Green master mix:

*GAPDH*_forward 5′-AAGGTGAAGGTCGGAGTCAA-3′;

*GAPDH*_reverse 5′-AATGAAGGGGTCATTGATGG-3′;

*MAPK14*_forward 5′-TGGAGAGCTTCTTCACTGCC-3′;

*MAPK14*_reverse 5′-CGAGCGTTACCAGAACCTGT-3′

### ROS measurement

Respectively 2.5.10^5^ and 5.10^5^ OCI-M1 and OCI-M2 cells per well were seeded in a 6-well plate in 2 ml medium with or without 5 μM AZD1208. Following 48 hours of culture, positive and negative control cells were treated according to the manufacturer's instructions with 400 μM tert-butyl hydroperoxide (TBHP) and 5 mM N-acetyl cysteine (NAC). All samples were incubated for 1 hour with 500 nM CellROX green (ROS indicator, Molecular Probes) or 5 μM MitoSOX red (mitochondrial superoxide indicator, Molecular Probes) and subsequently analyzed by flow cytometry using 488 nm excitation for CellROX green and 510 nm excitation for MitoSOX red.

### CellTiter-blue viability assay

Respectively 400 (OCI-M1), 500 (EOL-1), 1000 (U2932, K562, CMK), 2500 (OCI-M2 and TMD8), or 5000 (MOLM-16, KG1a) cells per well were seeded in a 96-well plate. After 24 hours, drugs were added to the medium in 2-fold serial dilutions using a HP Direct Digital Dispenser. After 5 days of culture CellTiter-Blue (Promega) was added. The conversion of resazurin into resorufin was measured by using an EnVision Multilabel Reader. Treatment with 10 μM phenyl arsenic oxide - resulting in complete cell death - was used as a baseline for viability.

### Growth assays using primary AML *ex vivo* cultures

#### MTT assay (Rotterdam)

AML patient samples were obtained at diagnosis from bone marrow or peripheral blood of patients who had given informed consent according to institutional guidelines. Based on the g­­ene expression profile, primary AML samples expressing high *PIM1* levels were selected for further experiments.

Patient samples, previously frozen in liquid nitrogen in the presence of 10% DMSO, were thawed and suspended in RPMI 1640 supplemented with 20% FCS, 1% insulin-transferrin-sodium selenite media supplement (Sigma-Aldrich), 1% L-glutamine, and 2% gentamicin. All samples contained 90% leukemic cells, determined morphologically on May-Grünwald-Giemsa (Merck)-stained cytospins.

Cells were plated in 96-well plates (1.5.10^5^ cells/well) containing AZD1208 and/or SCIO-469. After 4 days of culture, 3- [4,5-dimethylthiazol-2-yl]-2,5-diphenyl tetrazoliumbromide (MTT, 5 mg/ml, Sigma Aldrich) was added and the plates were incubated for an additional 6 hours. During this incubation, the MTT tetrazolium salt is reduced to purple-blue formazan crystals by viable cells. Crystals were dissolved by acidified isopropanol (0.04N HCl-isopropyl alcohol) and the optical density (OD) was measured at 562nm (VersaMax, Molecular Devices). Cell viability for each sample was furthermore measured at 0 hours to monitor cell growth during the 96 hours of culture.

It is important to note that a much larger number of cells derived from primary samples compared to cell lines is used per well for viability experiments. This results in a T_0_ read-out value that is much higher than PAO (whereas for cell line experiments these values are almost equal). Since this particular drug combination mainly acts cytostatic rather than cytotoxic, we used the T_0_ value as a baseline for viability in the presented graphs (y-axis = ‘0’). As a result, negative relative growth indicates a cytotoxic effect.

#### CellTiter-glo assay (Helsinki)

Bone marrow samples obtained from 5 diagnostic (samples 4325, 4361, 4368, 4374 and 4401) and 2 relapsed AML patients (samples 3853 and 370) with informed consent (approval # 239/13/03/00/2010, 303/13/03/01/2011) were applied for the drug testing experiments. Leukemic blast counts of the bone marrow samples were between 25 and 90%. Previously frozen mononuclear cells derived from these samples, isolated by Ficoll density gradient (Ficoll-Paque PREMIUM; GE Healthcare), were thawed and suspended in RPMI 1640 supplemented with 10% FCS, 12,5% human bone marrow stromal cell line HS-5 conditioned media [47], and 1% penicillin and streptomycin. 8000 cells per well were added in a 384 well plate containing AZD1208 and/or SCIO-469. After 72 hours of culture, CellTiter-Glo (Promega) reagent was added to the wells to measure ATP levels - the amount of ATP is proportional to the number of viable cells present. Luminescence was measured using a PHERAstar plate reader (BMG Labtech). Cell viability for each sample was furthermore measured at 0 hours to monitor cell growth during the 72 hours of culture.

It is important to note that a much larger number of cells derived from primary samples compared to cell lines is used per well for viability experiments. This results in a T_0_ read-out value that is much higher than PAO (whereas for cell line experiments these values are almost equal). Since this particular drug combination mainly acts cytostatic rather than cytotoxic, we used the T_0_ value as a baseline for viability in the presented graphs (y-axis = ‘0’). As a result, negative relative growth indicates a cytotoxic effect.

### Mouse xenografts

6- to 8-wk-old male Rag2^−/−^IL2γc^−/−^ mice were housed in a specific pathogen-free facility in individually ventilated cages at the Animal Core Facilities of the Center for Applied Medical Research (University of Navarra). All mouse experiments were performed in compliance with protocols approved by the local Animal Ethics Committee, which conform to institutional and national regulatory standards on experimental animal usage. K562 cells (5.10^6^) were subcutaneously implanted with matrigel (BD Bioscience) into the right flank of mice. When tumor size reached ~50 to 100 mm^3^, mice were randomly assigned and treated once daily with 30 mg/kg AZD1208 (AstraZeneca) by oral gavage and/or 5 mg/kg SB202190 (LC Laboratories) by intraperitoneal injection. Control group received vehicle (0.5% hydroxypropyl methylcellulose and 1% DMSO). All groups were composed of 11 to 18 mice. Tumor volume was measured three times per week with calipers and calculated as tumor volume = (length × width^2^) × 0.5.

### Synergy score calculation

Cells were seeded in a 96-well plate (384-well plate for Helsinki samples) and treated with 5*5 pairs (5*9 for Helsinki samples) of serially diluted drugs for the indicated number of days. Cell viability was measured using CellTiter-Blue (cell lines), MTT (Rotterdam samples), or CellTiter-Glo (Helsinki samples).

Measurements were normalized using ‘normalized percentage inhibition’ [48]. Values were transformed to values between 0 and 1 using the formula y = (x-p) /(n p) where x is the experimental value, n is the mean of the negative control, and p is the mean of the positive control (PAO). The effect level was calculated as: (1 - normalized value.) * 100 percent. A second matrix, the Loewe matrix, reflects the expected effect levels in case of additivity of the two drugs. The expected values were calculated using the formula of Loewe: D1/Dx1 + D2/Dx2 = 1 where D1 and D2 are the dose of respectively drug 1 and drug 2 in the combination. Dx1 is the single dose you would need from drug 1 in order to have the same effect x as the combination. Dx2 is this value for drug 2. Dx1 and Dx2 were determined using a fitted dose effect curve. The additive effect was determined with an heuristic method, in which for subsequent effect levels the outcome of the formula is calculated. The method starts with the highest effect of the two single drugs, for which the outcome of the formula will always be ≥ 1. The method continues until the value of 1 is crossed or the maximum of 100 is reached. The synergy score is calculated as the total of the positive values in the matrix divided by 100 [49]. Combination indices were calculated using the Chou-Talalay method [50]. Self-self synergy scores/ combination indices are the average score of all self-self drugs tested per cell line.

## SUPPLEMENTARY MATERIALS FIGURES


