# Supplementary material for: Intrinsic resistance to PIM kinase inhibition in AML through p38α-mediated feedback activation of mTOR signaling
Source: Oncotarget. 2016 Jun 5;7(25):37407–19. doi: 10.18632/oncotarget.9822 (PMC5122321; doi:10.18632/oncotarget.9822)
Supplement: Supplementary file 1 [file oncotarget-07-37407-s001.pdf]

Intrinsic resistance to PIM kinase inhibition in AML through p38α-mediated feedback activation of mTOR signaling

Supplementary Material

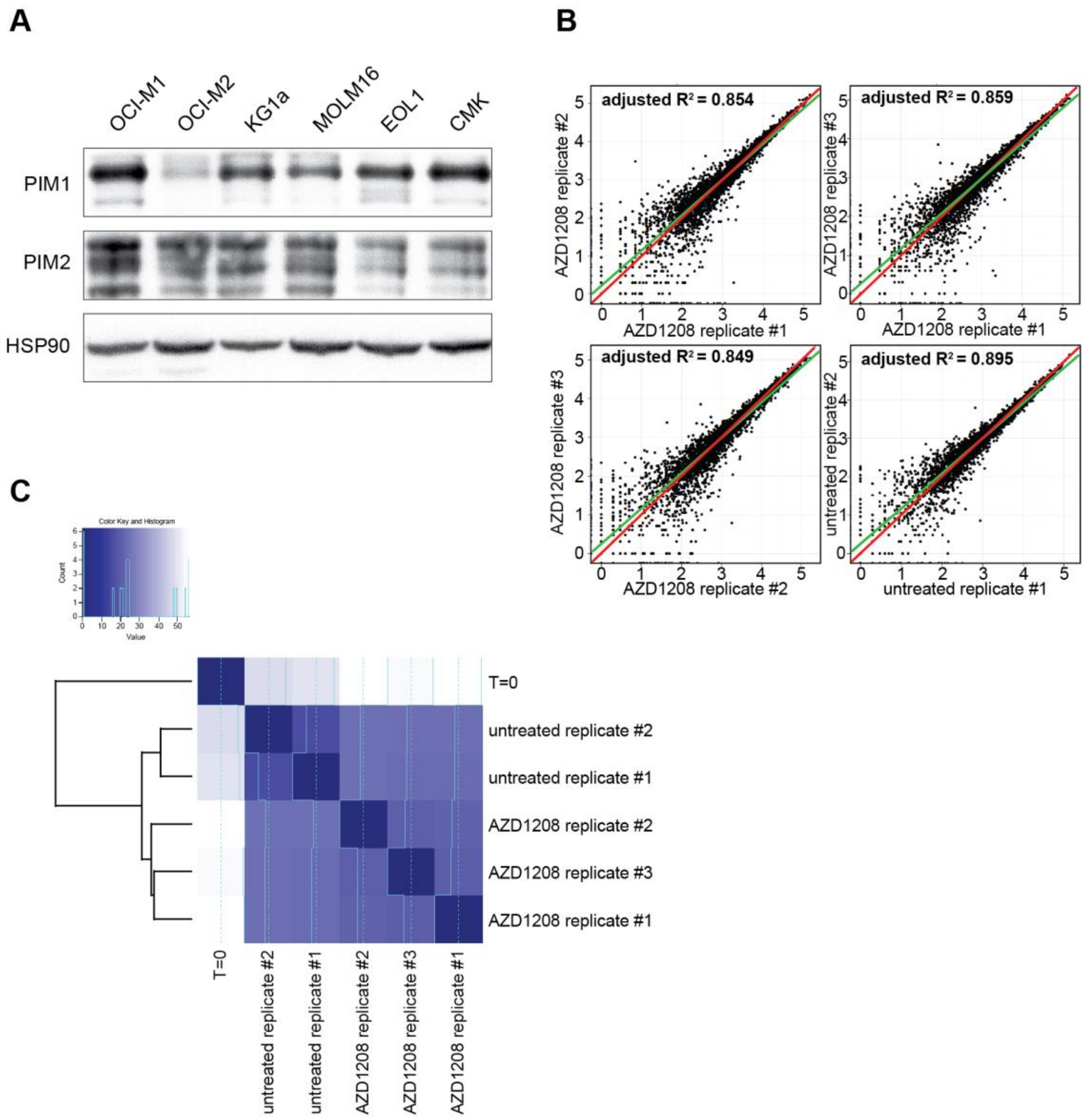

Supplemental Figure 1: Individual replicates of the shRNA screen demonstrate a high correlation. A. PIM1 and -2 protein levels in a panel of AML cell lines. **B**. Correlation graphs of replicates used in the shRNA screen. The x- and y-axis depict the number of sequence reads of each hairpin for the respective replicate. **C**. Cluster analysis of replicates used in the shRNA screen.

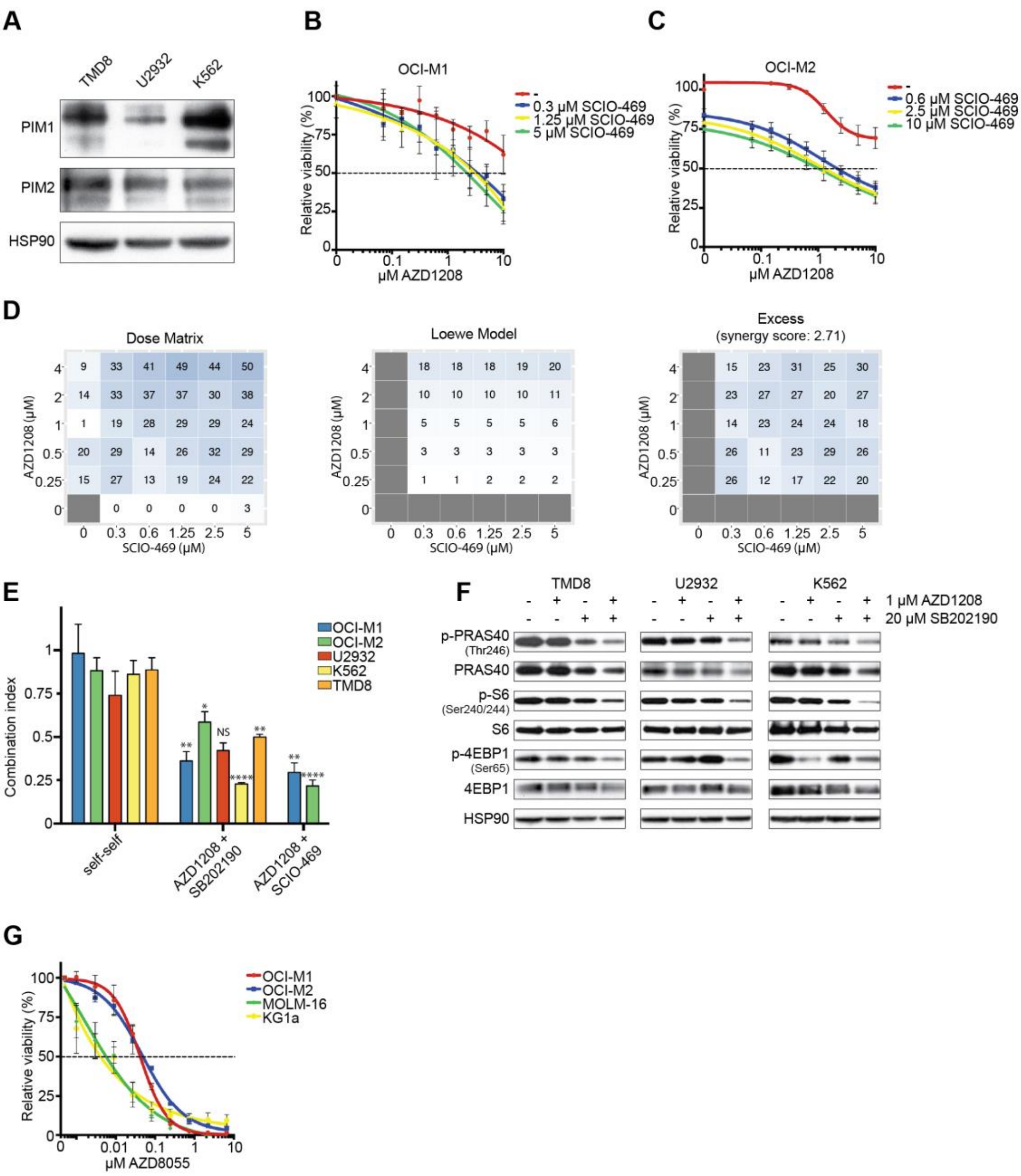

Supplemental Figure 2: Combined treatment of p38/PIM synergistically inhibits growth in cell lines. A. TMD8, U2932, and K562 express protein levels of PIM1 and -2. **B-C**. SCIO-469 enhances AZD1208 response in OCI-M1 and OCI-M2 cells. TMD8 cells were treated with increasing concentrations of AZD1208 (x-axis) and co-treated with the p38 inhibitor SCIO-469 (n=3). **D**. Example of a synergy score analysis. OCI-M1 cells were treated with increasing concentrations of AZD1208 (y-axis) and SCIO-469 (x-axis). The single and combined effects on growth inhibition are depicted in the dose matrix in percentages. The Loewe model is used to calculate the expected additive effect. The excess matrix represents the dose matrix minus the Loewe matrix. The synergy score is the sum of the positive differences divided by 100

(single agent excess scores are not included in calculation). Shown is a representative analysis. **E**. p38 inhibitors are synergistic with AZD1208. OCI-M1, OCI-M2, U2932, K562, and TMD8 cells were treated with 2-fold dilutions of AZD1208, SB202190, SCIO-469, or the combination for 5 days. Viability was assessed by CellTiter-Blue and used to calculate combination indices. Self-self combination treatments were used as a baseline to determine significance (n=3). P-values were calculated using a one-way ANOVA and Dunnett's test. $p \leq 0.05$ (*), $p \leq 0.01$ (**), and $p \leq 0.0001$ (****) **F**. Dual p38/PIM inhibition represses mTOR signaling. TMD8, U2932, and K562 cells were treated for 48 hours with 1 μM AZD1208, 20 μM SB202190, or the combination. Cell lysates were harvested and subjected to western blot analysis (n=2). **G**. PIM inhibitor resistant AML cell lines are sensitive to mTOR inhibition. OCI-M1, OCI-M2, MOLM16, and KG1a cells were treated with increasing concentrations of the mTOR inhibitor AZD8055 (x-axis). Viability was measured after 5 days using Cell-Titer-Blue (n=3)

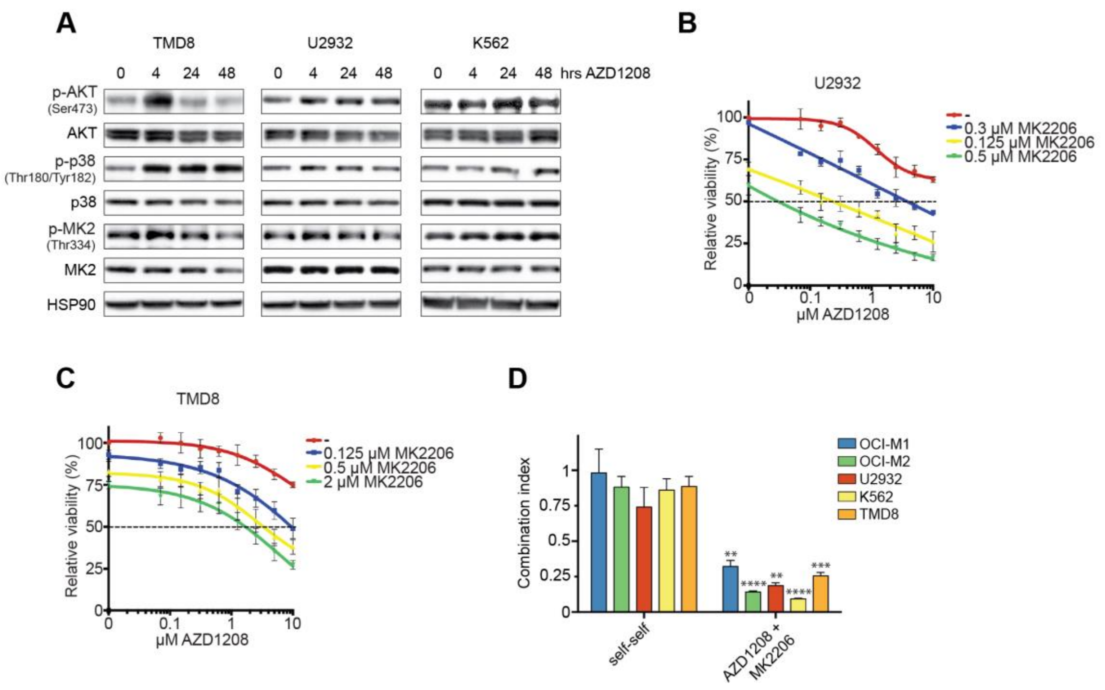

Supplemental Figure 3: AKT inhibition enhances the response to AZD1208. A. TMD8, U2932, and K562 cells were treated with 1 μM AZD1208 for 4, 24, and 48 hours. Cell lysates were harvested and subjected to western blot analysis. **B-C**. U2932 and TMD8 cells were treated with 2-fold dilutions of AZD1208, MK2206, or the combination for 5 days. Viability was assessed by CellTiter-Blue (n=3). **D**. Combination indices of combined AZD1208/MK2206 treatment. Self-self combination treatments were used as a baseline to determine significance (n=3). P-values were calculated using a one-way ANOVA and Dunnett's test. $p \leq 0.01$ (**), $p \leq 0.001$ (***), and $p \leq 0.0001$ (****)

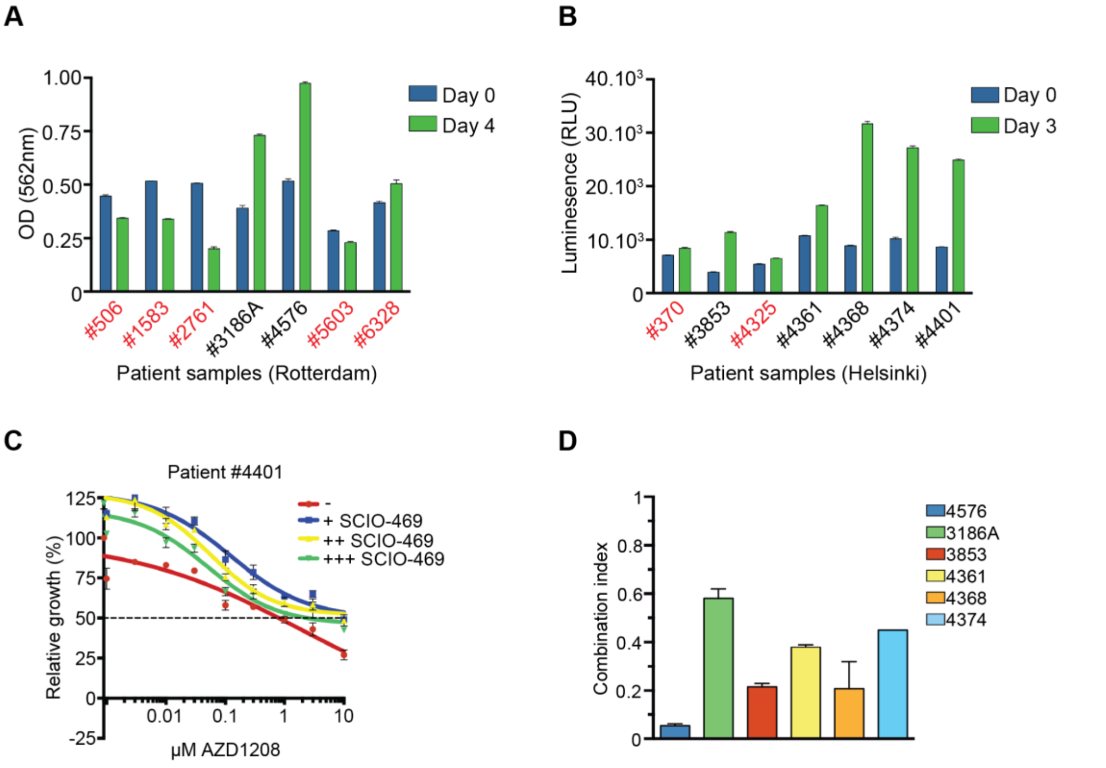

Supplemental Figure 4: Cell growth of patient samples. A. Optical density was measured at day 0 and day 4 to monitor cell growth. Only 2 out of 7 samples – depicted in black – (Rotterdam data set) demonstrated cell growth and were considered for further experiments. **B**. Luminescence was measured at day 0 and day 3 to monitor cell growth. 5 out of 7 samples – depicted in black – (Helsinki data set) demonstrated cell growth and were considered for further experiments. **C**. Patient #4401 cells were treated with increasing concentrations of AZD1208 (x-axis) and co-treated with 0.3 µM, 1 µM, or 3 µM SCIO-469. Viability was measured after 3 days using CellTiter-Glo. A day 0 measurement was used as a baseline value (y-axis = ‘0’) for growth. Negative relative growth indicates a cytotoxic effect (n=2). **D**. Combination indices for AZD1208 and SCIO-469 in *ex vivo* patient AML samples.
